# LncRNA OIP5-AS1 modulates the proliferation and apoptosis of Jurkat cells by sponging miR-181c-5p to regulate IL-7 expression in myasthenia gravis

**DOI:** 10.7717/peerj.13454

**Published:** 2022-05-17

**Authors:** Xu Wang, Huixue Zhang, Xiaoyu Lu, Shuang Li, Xiaotong Kong, Li Liu, Lifang Li, Si Xu, Tianfeng Wang, Jianjian Wang, Lihua Wang

**Affiliations:** Department of Neurology, The Second Affiliated Hospital of Harbin Medical University, Harbin, Heilongjiang, China

**Keywords:** Competing endogenous RNA (ceRNA), Myasthenia gravis (MG), OIP5-AS1, miR-181c-5p, IL-7

## Abstract

**Background:**

Myasthenia gravis (MG) is an antibody-mediated autoimmune disease. In recent years, accumulating evidence has indicated that long non-coding RNAs (lncRNAs) can function as competing endogenous RNAs (ceRNAs), contributing to the progression of various autoimmune diseases. Nevertheless, the regulatory roles of ceRNAs in MG pathogenesis remain unclear. In this study, we aimed to elucidate the role of lncRNA OIP5-AS1 as a ceRNA associated with MG progression.

**Methods:**

Real-time PCR was used to detect OIP5-AS1 levels in peripheral blood mononuclear cells (PBMCs) from patients with MG. Luciferase reporter assays were performed to validate the relationship between OIP5-AS1 and miR-181c-5p. CCK-8 and flow cytometry were performed to test the proliferation and apoptotic abilities of OIP5-AS1 in Jurkat cells. Furthermore, real-time PCR and Western blot assays were performed to explore the interactions between OIP5-AS1, miR-181c-5p, and IL-7.

**Results:**

The expression of OIP5-AS1 was up-regulated in patients with MG. Luciferase reporter assay indicated that OIP5-AS1 targeted the miR-181c-5p. Functional assays showed that OIP5-AS1 suppressed Jurkat cell apoptosis and promoted cell proliferation by sponging miR-181c-5p. Mechanistically, knockdown of OIP5-AS1 inhibited IL-7 expression at both the mRNA and protein levels in Jurkat cells, whereas the miR-181c-5p inhibitor blocked the reduction of IL-7 expression induced by OIP5-AS1 suppression.

**Conclusions:**

We confirmed that OIP5-AS1 serves as an endogenous sponge for miR-181c-5p to regulate the expression of IL-7. Our findings provide novel insights into MG processes and suggests potential therapeutic targets for patients with MG.

## Introduction

Myasthenia gravis (MG) is an antibody-mediated autoimmune disease of the postsynaptic membrane of the neuromuscular junction (Gilhus et al., 2019). Studies have shown that most patients with MG had antibodies against the acetylcholine receptor (AChR), and a small subgroup of patients had antibodies against muscle-specific tyrosine kinase (MuSK) or lipoprotein-receptor-related protein 4 (LRP4) (Cortés-Vicente et al., 2016). These antibodies induce defects in neuromuscular transmission with fatigable muscle weakness (Gilhus, 2016; Vincent, Palace & Hilton-Jones, 2001). Furthermore, many pro-inflammatory cytokines produced by immune cells are important regulators of MG development. For example, it has been reported that IL-21, IL-4, and IL-17A were highly expressed in MG patients and associated with its pathogenesis (Çebi et al., 2020). However, the molecular mechanisms underlying the pathogenesis of MG remains unclear.

Long non-coding RNAs (lncRNAs) are more than 200 nucleotides in length, with limited protein-coding capacity (Fatica & Bozzoni, 2014). In addition, lncRNAs regulate protein-coding gene expression through interactions with DNA, chromatin, RNA, and proteins (Chen, Satpathy & Chang, 2017). Previous studies have shown that lncRNAs play an important role in MG progression. For example, lncRNA SNHG16 is up-regulated in MG patients and promotes MG progression by targeting the let-7c-5p/IL-10 axis (Wang et al., 2020a). MicroRNAs (miRNAs) are small non-coding RNAs that regulate gene expression at the post-transcriptional level by directly targeting the 3′-untranslated region (3′-UTR) of genes (Long et al., 2018). Several miRNAs have been identified as important regulators of MG pathogenesis. For instance, miR-320a is downregulated in MG patients and induces the overexpression of pro-inflammatory cytokines by targeting MAPK1 (Cheng et al., 2013).

In recent years, the interaction between lncRNAs and miRNAs in the pathogenesis of MG has garnered increasing attention. Furthermore, lncRNAs have been reported to act as competitive endogenous RNAs (ceRNAs) to regulate the expression of target genes by sponging miRNAs (Tay, Rinn & Pandolfi, 2014). Moreover, several ceRNA networks have been identified in various diseases. For example, HOTAIR plays an important role in gastric cancer by acting as a ceRNA that sponges miR-331-3p to regulate HER2 expression (Liu et al., 2014). UCA1 is up-regulated in renal cancer and regulates DLL4 expression by sponging miR-182-5p (Wang et al., 2020b). Opa-interacting protein 5 antisense RNA 1 (OIP5-AS1) is an lncRNA involved in cell proliferation, migration, invasion, and apoptosis in colorectal cancer (Wang, Lin & Liu, 2021), endometrial carcinoma (Liang et al., 2021), osteosarcoma (Dai et al., 2018), and hepatocellular carcinoma (Wang et al., 2019). In addition, OIP5-AS1 expression is elevated in patients with multiple sclerosis (MS) and promotes MS progression by targeting the miR-140-5p/RhoA axis (Liu et al., 2021). Therefore, while OIP5-AS1 may be considered a potential biological marker for autoimmune diseases, its expression levels and biological functions in MG remain unclear.

In this study, we investigated the expression of OIP5-AS1 in peripheral blood mononuclear cells (PBMCs) of patients with MG. Moreover, whether OIP5-AS1 regulates miRNA function in MG remains to be clarified. Therefore, the present study was designed to determine whether OIP5-AS1 mediates the progression of MG by regulating IL-7 by sponging miR-181c-5p.

## Materials and Methods

### Clinical samples

Thirty-four patients diagnosed with MG (15 men, 44.1%) from the Second Affiliated Hospital of Harbin Medical University were enrolled in this study. The average age of the experimental group was 55.74 ± 16.85 years. In addition, 30 sex- and age-matched healthy controls (13 men, 43.33%) with no history of autoimmune disease were included. The average age of the control group was 55.00 ± 13.20 years. This study was approved by the Ethics Committee of the Second Affiliated Hospital of Harbin Medical University (ky2020-105). Written informed consent was obtained from all the patients and healthy controls. Approximately 5 ml of peripheral blood from each subject was collected into EDTA-supplemented tubes. PBMCs were isolated by density-gradient centrifugation over Ficoll-Hypaque solution (Haoyang Biological Technology Co., Tianjin, China) and stored at −80 °C.

### Cell culture

Jurkat T-cell leukemia cell line and human embryonic kidney epithelial cell line HEK293T were purchased from the American Type Culture Collection (Manassas, VA, USA). Jurkat cells were cultured in RPMI 1640 medium (Gibco, Billings, MT, USA), whereas HEK293T cells were cultured in Dulbecco’s modified Eagle medium (DMEM; Gibco, Billings, MT, USA). All media contained 10% fetal bovine serum (FBS; Gibco, Billings, MT, USA) and 1% penicillin/streptomycin (Invitrogen, Waltham, CA, USA). The cells were incubated at 37 °C in a humidified atmosphere containing 5% CO_2_.

### Cell transfection

Several components were procured for this assay, including short hairpin RNAs (shRNA) targeting OIP5-AS1 (shOIP5-AS1) and its negative control (shRNA-NC), miR-181c-5p mimics and its negative control, and miR-181c-5p inhibitor and its negative control were designed and synthesized by GenScript (Nanjing, China). All sequences are listed in Table 1. Before transfection, the cells were seeded into six-well plates and cultured for 24 h. When cell confluence reached 60–80%, the plasmids were transfected into cells using the Lipofectamine™ 3000 Transfection Reagent (Invitrogen, Carlsbad, CA, USA) according to the manufacturer’s instructions. After transfection for 6 h, the culture supernatants of the transfected cells were replaced with fresh culture medium. Following this, real-time PCR analysis was performed to determine transfection efficiency, and the highest transfection rate was observed at 48 h post-transfection.

**Table 1:** Sequences of miRNA mimics, miRNA inhibitors, and shRNA.

Gene	Sequences (5′–3′)
miR-181c-5p mimics	Forward: AACAUUCAACCUGUCGGUGAGU
	Reverse: UCACCGACAGGUUGAAUGUUUU
mimics NC	Forward: UUCUCCGAACGUGUCACGUTT
	Reverse: ACGUGACACGUUCGGAGAATT
miR-181c-5p inhibitor	ACUCACGACAGGUUGAAUGUU
inhibitor NC	CAGUACUUUUGUGUAGUACAA
shOIP5-AS1-1	GAGGAGAGGAACTAACCGAATTCAAGAGATTCGGTTAGTTCCTCTCCTTTTTT
shOIP5-AS1-2	GTGGGTAGTCCTGCTTCTTTTTCAAGAGAAAAGAAGCAGGACTACCCATTTTT
shOIP5-AS1-3	GCTCCCAAGTAGCTAGGATTTTCAAGAGAAATCCTAGCTACTTGGGAGTTTTT
shRNA NC	GTTCTCCGAACGTGTCACGTTTCAAGAGAACGTGACACGTTCGGAGAATTTTT

**Note:**

miR-181c-5p, microRNA-181c-5p; OIP5-AS1, opa-interacting protein 5 antisense RNA 1; NC, negative control; sh, shRNA.

### Real-time PCR analysis

Total RNA was extracted from PBMCs or Jurkat cells using TRIzol™ Reagent (Invitrogen, Waltham, CA, USA). Next, total RNA (2 μg) was reverse-transcribed into complementary DNA (cDNA) using a Transcriptor First Strand cDNA Synthesis Kit (Roche Diagnostics). An miRcute Plus miRNA First-Strand cDNA Synthesis kit (Tiangen, Beijing, China) was used to synthesize miRNA cDNA from total RNA. Real-time PCR for OIP5-AS1 and IL-7 was performed using LightCycler^®^ 480 SYBR Green Master Mix (Roche Diagnostics, Basel, Switzerland) with an Applied Biosystems 7,500 Real-Time PCR system (Thermo Fisher Scientific, Waltham, MA, USA). The reaction conditions were as follows: initial denaturation at 95 °C for 3 min, followed by 40 cycles of denaturation at 95 °C for 15 s, annealing at 60 °C for 40 s, and elongation at 72 °C for 20 s and GAPDH was used as an internal control. Real-time PCR for miR-181c-5p was performed using an miRcute Plus miRNA qPCR Detection Kit (Tiangen, Beijing, China), which contained both the specific reverse primers of miRNAs and the internal control U6. The reaction was performed at 95 °C for 15 min, followed by 40 cycles of 20 s at 94 °C and 34 s at 60 °C. The relative expression of the target genes was expressed as GAPDH/U6 relative fold and calculated using the 2^−ΔΔCT^ method. The primer sequences used are listed in Table 2.

**Table 2:** Primer sequences.

Gene	Primer	Sequences
OIP5-AS1	Forward Primer (5′–3′)	TGTATTAGCCCTGCTCGTT
	Reverse Primer (5′–3′)	TGCAAATCCTGGTCCATC
IL-7	Forward Primer (5′–3′)	TTCCATGTTTCTTTTAGGT
	Reverse Primer (5′–3′)	TATTGTTTGCCATCTTTAC
GAPDH	Forward Primer (5′–3′)	GACCTGACCTGCCGTCTAG
	Reverse Primer (5′–3′)	AGGAGTGGGTGTCGCTGT
miR-181c-5p	Forward Primer (5′–3′)	AACATTCAACCTGTCGGTGAGT
U6	Forward Primer (5′–3′)	CTCGCTTCGGCAGCACA

**Note:**

OIP5-AS1, opa-interacting protein 5 antisense RNA 1; IL-7, Interleukin 7; miR-181c-5p, microRNA-181c-5p; GAPDH, glyceraldehyde-3-phosphate dehydrogenase.

### Western blot analysis

The total protein isolated from Jurkat cells were separated using radioimmunoprecipitation assay (RIPA) buffer (Beyotime Biotechnology, Shanghai, China). The protein concentration was determined using a bicinchoninic acid protein assay kit (Wanleibio, Shenyang, China). Furthermore, proteins were separated using sodium dodecyl sulfate-polyacrylamide gel electrophoresis (SDS-PAGE; Wanleibio, Shenyang, China) and electrophoretically transferred to polyvinylidene fluoride (PVDF) membranes (Millipore, Billerica, MA, USA). Subsequently, they were blocked with 5% nonfat milk for 1 h at room temperature and incubated overnight at 4 °C with IL-7 (1:1000; ABclonal, Wuhan, China) and GAPDH primary antibodies (1:1000; Wanleibio, Beijing, China). The membranes were washed with Tris-buffered saline (TBS) and Tween-20 (TBST) and incubated with goat anti-rabbit secondary antibodies for 2 h at room temperature. Subsequently, the protein blots were visualized using an enhanced chemiluminescence reagent (ECL; Wanleibio, Beijing, China), while GAPDH was used as the internal reference. The intensity was quantified using the ImageJ software (National Institutes of Health, Bethesda, MD, USA).

### Luciferase reporter assay

Fragments containing the wild-type (WT) and mutant-type (MUT) of the lncRNA OIP5-AS1 fragment were constructed into a pmirGLO Dual-Luciferase Target Expression Vector (GenScript, Nanjing, China). HEK293T cells were co-transfected with the OIP5-AS1-WT/OIP5-AS1-MUT vectors and miR-181c-5p mimic/miRNA NC using Lipofectamine 3000 (Invitrogen, Waltham, MA, USA). Luciferase activity was measured using the Dual-Luciferase Reporter Assay Kit (Promega, Madison, WI, USA) according to the manufacturer’s instructions. Relative luciferase activity was measured and normalized to Renilla luciferase activity at 48 h post-transfection. The transfection assays were performed in triplicate.

### Cell proliferation assay

The Cell Counting Kit-8 (CCK-8; Wanleibio, Shenyang, China) was used to detect cell viability according to the manufacturer’s instructions. Jurkat cells were seeded in 96-well plates at a density of 6 × 10^3^ cells/well. After transfection with the negative control, shOIP5-AS1, and shOIP5-AS1 along with miR-181c-5p inhibitor and incubation for 24, 48, 72, and 96 h, each well was added with 10 μl CCK-8 solution and cultured for an additional 2 h at 37 °C. Optical density at 450 nm was measured using a microplate reader (Bio-Tek Instruments, Winooski, VT, USA). The experiments were independently repeated thrice.

### Apoptosis analysis

Jurkat cells were transfected with the negative control, shOIP5-AS1, and shOIP5-AS1 along with the miR-181c-5p inhibitor and cultured in a six-well plate. After 48 h of incubation at 37 °C, the apoptosis assay was performed using the Annexin V-Light 650/PI Apoptosis Detection Kit (Wanleibio, Shenyang, China). After centrifugation, the cells were collected and resuspended in 500 μl of binding buffer and incubated with 5 μl of Annexin V-light 650 and 10 μl of propidium iodide (PI) for 15 min in the dark. A flow cytometer was used to detect apoptosis at an excitation wavelength of 488 nm. Each experiment was independently repeated in triplicates.

### Statistical analysis

SPSS 23.0 software was utilized for statistical analyses. All data are expressed as the mean ± standard deviation (SD). Differences between the two groups were evaluated using the Student’s *t*-test. The differences between three or more groups were analyzed using one-way analysis of variance (ANOVA), and Tukey’s *post hoc* test was used for pairwise comparisons after one-way ANOVA. The correlation between the expression of OIP5-AS1, miR-181c-5p, and IL-7 in patients with MG was evaluated using Pearson’s correlation analysis. Statistical significance was set at *p* < 0.05.

## Results

### LncRNA OIP5-AS1 is up-regulated, and miR-181c-5p is downregulated in MG patients

**Figure 1:**
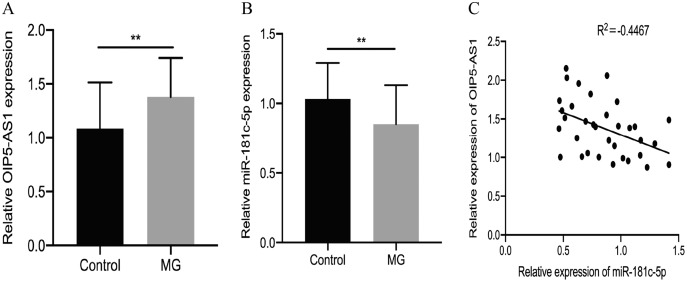
LncRNA OIP5-AS1 is up-regulated, and miR-181c-5p is downregulated in MG patients. (A) OIP5-AS1 expression was examined in MG patients and control subjects by real-time PCR. (B) miR-181c-5p expression was examined in MG patients and control subjects by real-time PCR. (C) Pearson’s correlation was performed to analyze the correlation between OIP5-AS1 and miR-181c-5p expression in PBMCs of MG patients. The experiment was repeated at least three times, and the data are presented as the mean ± SD. **p < 0.01.

We used real-time PCR analysis to determine whether OIP5-AS1 was involved in the progression of MG. The results showed that OIP5-AS1 was up-regulated in PBMCs of patients with MG compared to healthy controls (*p* = 0.0041, Fig. 1A). Bioinformatics analysis (starBase v3.0) revealed that the OIP5-AS1 sequence contains a potential binding site for miR-181c-5p. A previous study found that miR-181c-5p is downregulated in MG patients (Zhang et al., 2016). Consistent with previous results, we subsequently verified that miR-181c-5p was downregulated in PBMCs of patients with MG compared to healthy controls (*p* = 0.0091, Fig. 1B). In addition, Pearson’s correlation analysis suggested a negative correlation between OIP5-AS1 and miR-181c-5p in the PBMCs of MG patients (*p* = 0.0081, Fig. 1C).

### OIP5-AS1 is a direct target of miR-181c-5p

**Figure 2:**
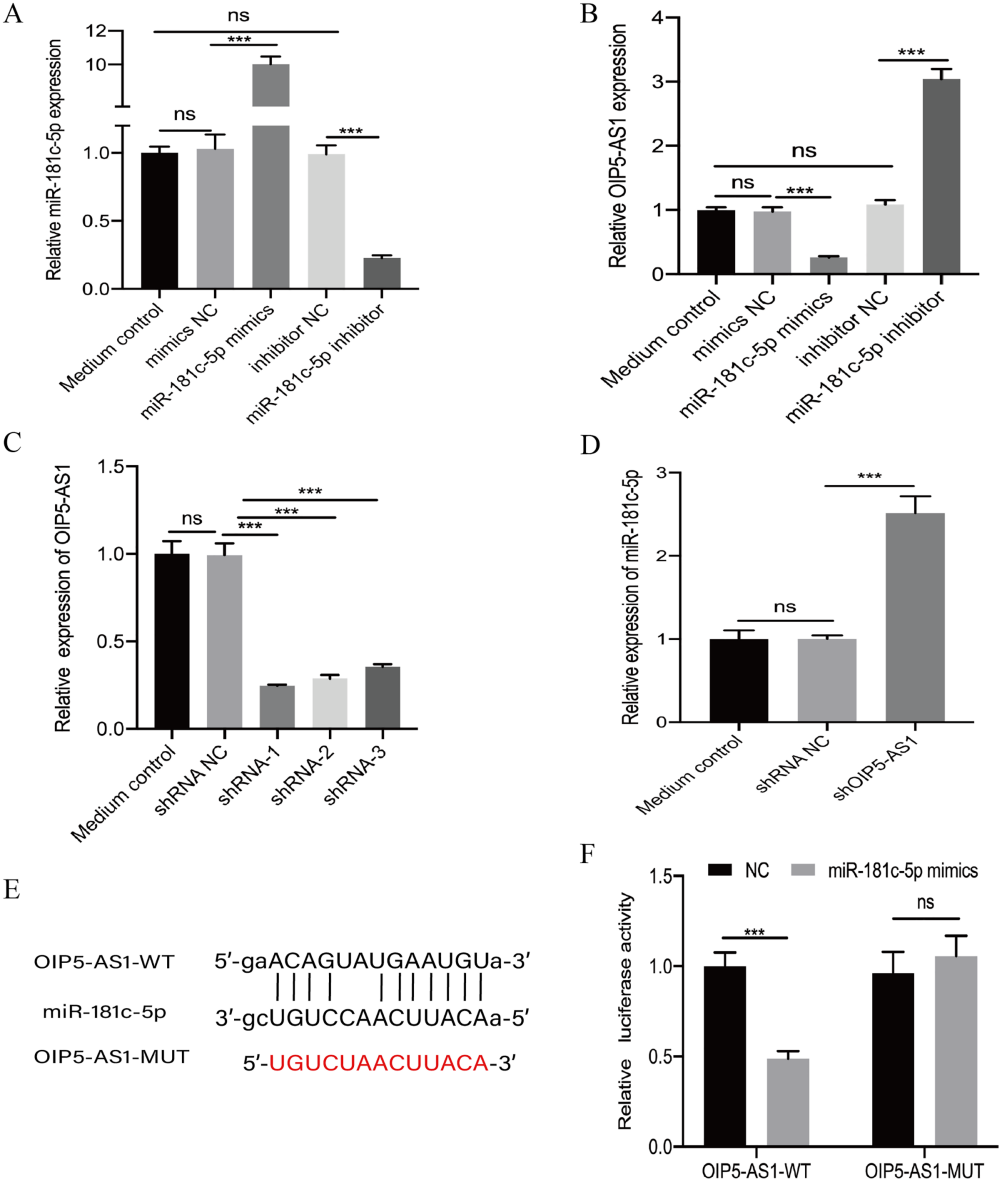
OIP5-AS1 is a direct target of miR-181c-5p. (A) The transfection efficiency of miR-181c-5p mimics and miR-181c-5p inhibitor were measured by real-time PCR. (B) The relative expression level of OIP5-AS1 in Jurkat cells transfected with miR-181c-5p mimics or miR-181c-5p inhibitor was measured using real-time PCR. (C) The expression of OIP5-AS1 in Jurkat cells transfected with shRNA against OIP5-AS1 (shRNA-1, shRNA-2, and shRNA-3), or negative control (shRNA NC) was measured using real-time PCR. The shRNA-1 with better knockdown efficiency was used for subsequent experiment, named shOIP5-AS1. (D) The relative expression level of miR-181c-5p in Jurkat cells transfected with shRNA NC or shOIP5-AS1 was measured using real-time PCR. (E) The putative miR-181c-5p binding sequence of the wide-type and mutation sequence of OIP5-AS1. (F) The luciferase reporter plasmid containing OIP5-AS1-WT or OIP5-AS1-MUT was co-transfected with miR-181c-5p mimics or miRNA NC into HEK293T cells. The experiment was repeated at least three times, and the data are presented as the mean ± SD. ***p < 0.001. ns, no significant.

We further studied the relationship between OIP5-AS1 and miR-181c-5p. The miR-181c-5p mimic and its negative control, miR-181c-5p inhibitor, and its negative control were transfected into Jurkat cells. The successful transfection efficiency of miR-181c-5p mimics and miR-181c-5p inhibitor was identified at the mRNA level using real-time PCR (*p* < 0.001, Fig. 2A). The results indicated that miR-181c-5p mimics reduced the expression of OIP5-AS1, whereas the miR-181c-5p inhibitor increased the expression of OIP5-AS1 in Jurkat cells (*p* < 0.001, Fig. 2B). We transfected Jurkat cells with three shRNAs against OIP5-AS1 and a negative control (shRNA-NC). Transfection with shOIP5-AS1-1 exhibited the highest knockdown efficiency compared to shOIP5-AS1-2 and shOIP5-AS1-3 and was thus chosen for subsequent experiments (*p* < 0.001, Fig. 2C). Real-time PCR results showed that knockdown of OIP5-AS1 significantly increased miR-181c-5p expression compared to that in the negative control group (*p* < 0.001, Fig. 2D). A dual-luciferase reporter assay was performed to further explore the correlation between miR-181c-5p and OIP5-AS1. The OIP5-AS1-WT and OIP5-AS1-MUT luciferase reporter vectors were constructed (Fig. 2E). They were then co-transfected with miR-181c-5p mimics and negative control into HEK293T cells. The results showed that miR-181c-5p mimics markedly reduced the luciferase activity of OIP5-AS1-WT; however, the luciferase activity of OIP5-AS1-MUT remained unchanged (*p* < 0.001, Fig. 2F). These results indicate that OIP5-AS1 competitively binds to miR-181c-5p.

### OIP5-AS1 acts as a ceRNA by sponging miR-181c-5p to modulate IL-7 expression

**Figure 3:**
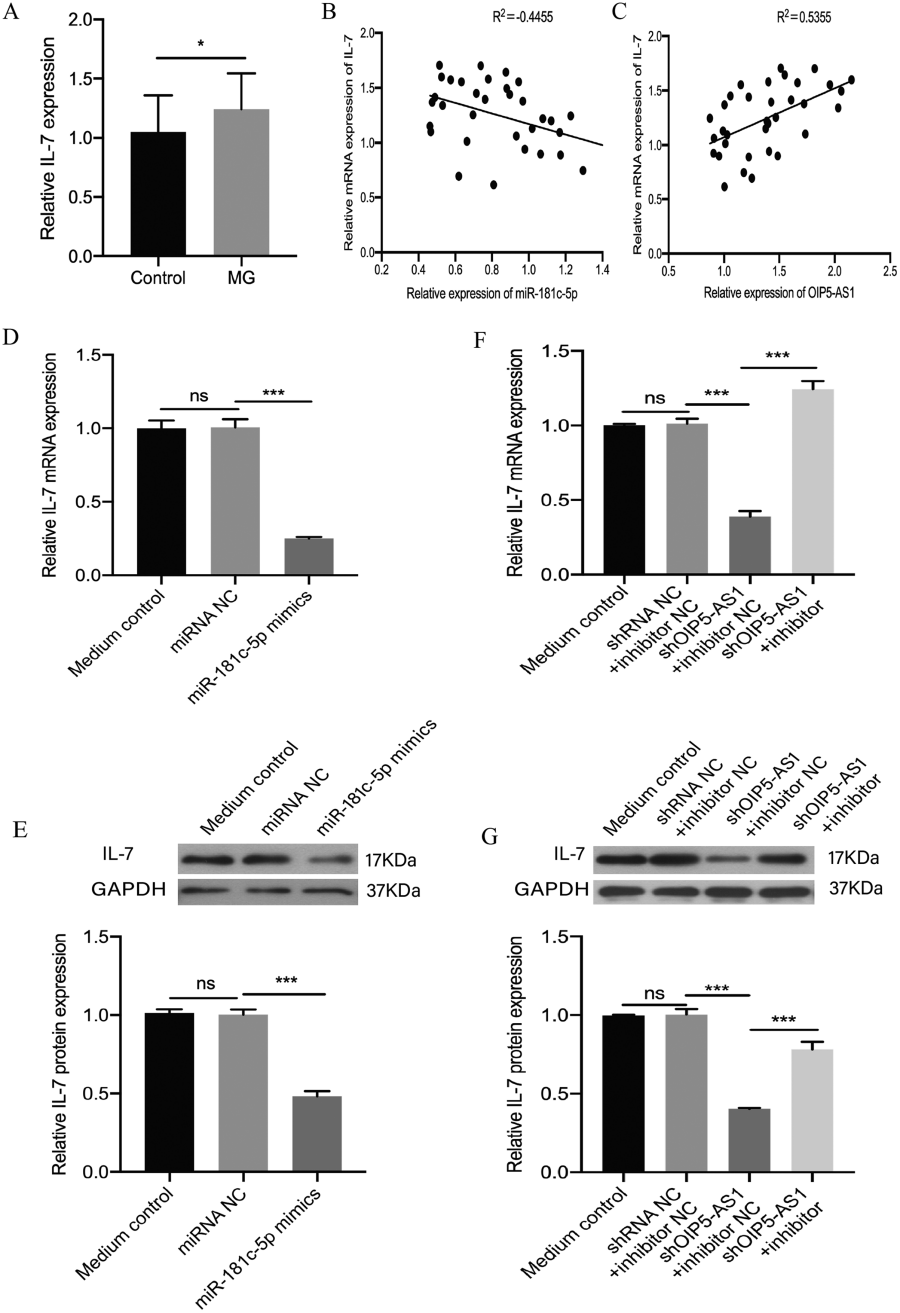
OIP5-AS1 regulates IL-7 expression by sponging miR-181c-5p in a ceRNA manner. (A) IL-7 expression was examined in MG patients and control subjects by real-time PCR. (B) Pearson’s correlation was performed to analyze the correlation between IL-7 and miR-181c-5p expression in PBMCs of MG patients. (C) Pearson’s correlation was performed to analyze the correlation between IL-7 and OIP5-AS1 expression in PBMCs of MG patients. (D) Relative expression levels of IL-7 mRNA were measured by real-time PCR after transfection with miR-181c-5p mimic or miRNA NC in Jurkat cells. (E) Relative expression levels of IL-7 protein were measured by western blotting after transfection with miR-181c-5p mimic or miRNA NC in Jurkat cells. (F) Relative expression levels of IL-7 mRNA were measured by real-time PCR after transfection with shRNA NC + miR-181c-5p inhibitor NC, shOIP5-AS1 + miR-181c-5p inhibitor NC, and shOIP5-AS1 + miR-181c-5p inhibitor in Jurkat cells. (G) Relative expression levels of IL-7 protein were measured by western blotting after transfection with shRNA NC + miR-181c-5p inhibitor NC, shOIP5-AS1 + miR-181c-5p inhibitor NC, and shOIP5-AS1 + miR-181c-5p inhibitor in Jurkat cells. The experiment was repeated at least three times, and data are presented as the mean ± SD. *p < 0.05, ***p < 0.001. ns, no significant.

Furthermore, previous studies showed that IL-7 is a target of miR-181c-5p (Zhang et al., 2016). In our study, real-time PCR results confirmed that the expression level of IL-7 was up-regulated in the PBMCs of patients with MG (*p* = 0.0139, Fig. 3A). Moreover, the correlation between the expression of OIP5-AS1, miR-181c-5p, and IL-7 was determined. We found that the expression of IL-7 and miR-181c-5p in MG patients was negatively correlated (*p* = 0.0083, Fig. 3B), whereas the expression of IL-7 and OIP5-AS1 in MG patients was positively correlated (*p* = 0.0011, Fig. 3C). We investigated whether OIP5-AS1 modulates IL-7 expression by sponging miR-181c-5p. Jurkat cells were transfected with the miR-181c-5p mimic and a negative control. Real-time PCR and Western blotting results showed that overexpression of miR-181c-5p repressed IL-7 expression at both the mRNA and protein levels (*p* < 0.001, Figs. 3D and 3E). Jurkat cells were transfected with the negative control, shOIP5-AS1, or shOIP5-AS1 in combination with miR-181c-5p inhibitor. The results revealed that knockdown of OIP5-AS1 significantly reduced IL-7 mRNA and protein expression levels, while the miR-181c-5p inhibitor nullified the decrease in IL-7 expression induced by OIP5-AS1 suppression (*p* < 0.001, Figs. 3F and 3G). These findings suggest that OIP5-AS1 regulated the expression of IL-7 by sponging miR-181c-5p in a ceRNA manner.

### OIP5-AS1 inhibits apoptosis and promotes proliferation by competing with miR-181c-5p in Jurkat cells

**Figure 4:**
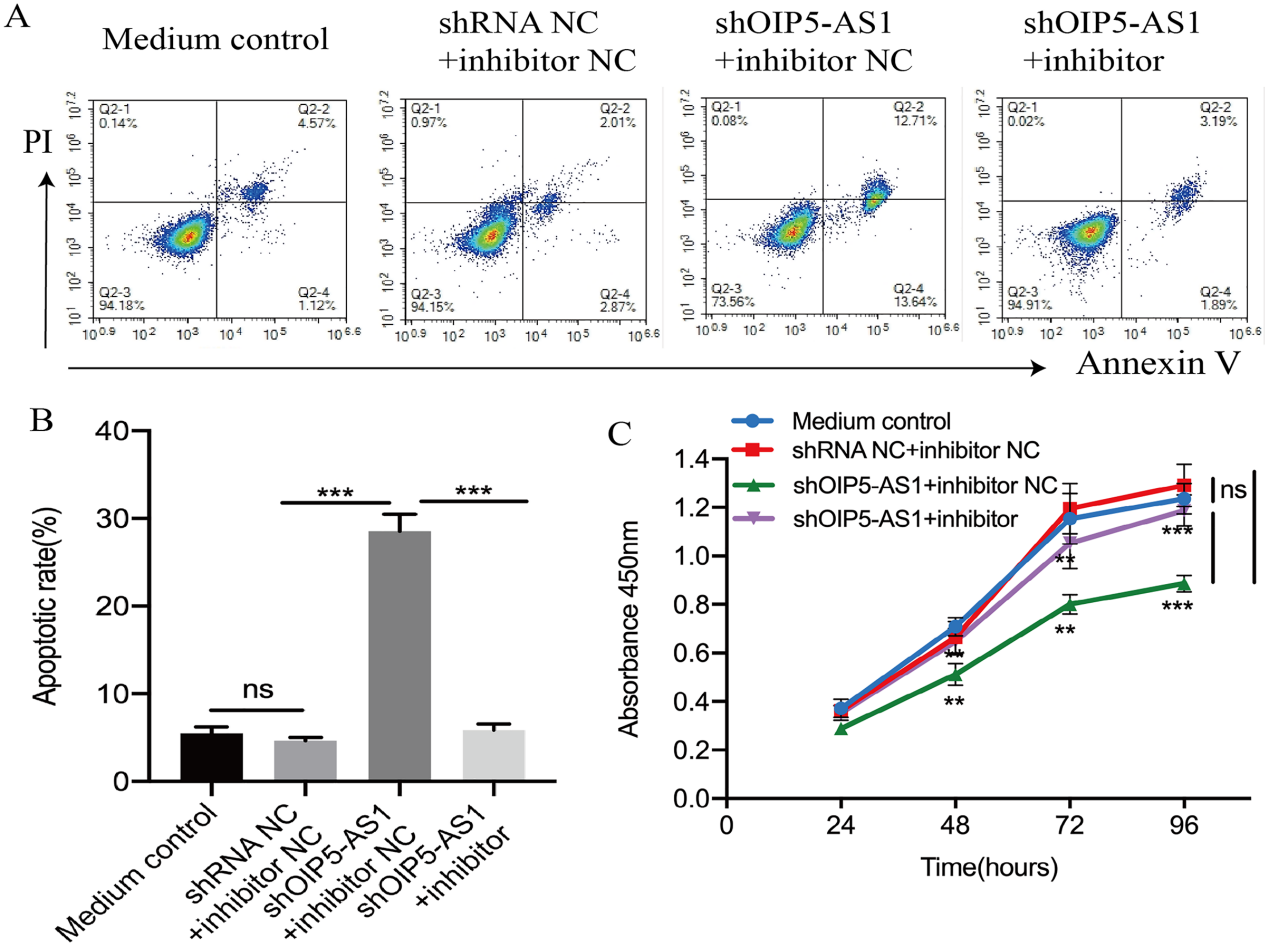
OIP5-AS1 inhibits apoptosis and promotes cell proliferation by sponging miR-181c-5p. (A) Flow cytometry was performed to determine cell apoptosis after transfection with shRNA NC + miR-181c-5p inhibitor NC, shOIP5-AS1 + miR-181c-5p inhibitor NC, and shOIP5-AS1 + miR-181c-5p inhibitor for 48 h into Jurkat cells. (B) The apoptosis rate of Jurkat cells after transfection with shRNA NC + miR-181c-5p inhibitor NC, shOIP5-AS1 + miR-181c-5p inhibitor NC, and shOIP5-AS1 + miR-181c-5p inhibitor. (C) CCK-8 assay was performed to determine cell proliferation after transfection with shRNA NC + miR-181c-5p inhibitor NC, shOIP5-AS1 + miR-181c-5p inhibitor NC, and shOIP5-AS1 + miR-181c-5p inhibitor into Jurkat cells. The experiment was repeated at least three times, and data are presented as the mean ± SD. **p< 0.01, ***p< 0.001. ns, no significant.

In order to explore the OIP5-AS1/miR-181c-5p mechanism in Jurkat cells, flow cytometry and CCK-8 assays were performed to assess the rates of apoptosis and proliferation. Jurkat cells were transfected with the negative control, shOIP5-AS1, and shOIP5-AS1 along with the miR-181c-5p inhibitor. Flow cytometry results indicated that knockdown of OIP5-AS1 induced Jurkat cell apoptosis compared to the negative control group, and this effect was reversed by transfecting with the miR-181c-5p inhibitor (*p* < 0.001, Figs. 4A and 4B). Furthermore, the CCK-8 assay indicated that OIP5-AS1 inhibition reduced Jurkat cell proliferation compared to the negative control group, whose proliferation was reversed by co-transfection with the miR-181c-5p inhibitor (*p* < 0.001, Fig. 4C). The apoptosis and proliferation of Jurkat cells in the medium and negative control groups exhibited no significant differences. Altogether, these results suggest that OIP5-AS1 inhibits apoptosis and promotes proliferation *via* sponging miR-181c-5p in Jurkat cells.

## Discussion

MG is an autoimmune disease characterized by an antibody-mediated disorder of neuromuscular junction transmission (Romi, Gilhus & Aarli, 2005). However, its immunological pathogenesis has not been clearly defined. Some immunosuppressive drugs have proven effective in the treatment of MG patients by interfering with B and T cells, but the treatment is not immunospecific (Mantegazza, Bernasconi & Cavalcante, 2018). Therefore, there is an urgent need to investigate the pathogenesis of MG and determine the optimal treatment for individual patients. Recent studies have shown that lncRNAs can regulate gene expression at the transcriptional and post-transcriptional levels. A growing number of lncRNAs have been identified as important regulators of the development of autoimmune diseases (Statello et al., 2021). For example, lncRNA PICSAR is up-regulated in rheumatoid arthritis (RA) and is involved in the pathogenesis of fibroblast-like synoviocytes by sponging miRNA-4701-5p (Bi et al., 2019). However, very few lncRNAs undergo dysregulation in MG (Luo et al., 2017; Niu et al., 2020). Therefore, it is necessary to identify more abnormally expressed lncRNAs during MG development. In our study, we first demonstrated that OIP5-AS1 was up-regulated in PBMCs of patients with MG. Knockdown of OIP5-AS1 significantly inhibited the proliferation and promoted the apoptosis of Jurkat cells. Therefore, we hypothesized that OIP5-AS1 was involved in MG pathogenesis.

Accumulating evidence has validated that lncRNAs, particularly those that exist in the cytoplasm, regulate the expression of miRNA target genes by acting as competing endogenous RNAs (ceRNAs) (Batista & Chang, 2013). For example, in osteosarcoma, OIP5-AS1 has been reported to serve as a ceRNA of miR-223 to regulate CDK14 expression and mediate osteosarcoma cell proliferation and apoptosis (Dai et al., 2018). Additionally, OIP5-AS1 acts as a ceRNA by binding to miR-3163 to up-regulate VEGFA expression in hepatocellular carcinoma (Shi et al., 2020). However, the role of OIP5-AS1 in MG development remains unclear. We speculated that OIP5-AS1 may function as a ceRNA in MG. Using bioinformatic analysis, we found that miR-181c-5p could be a potential target miRNA of OIP5-AS1. Our findings demonstrated that the expression of miR-181c-5p was downregulated in the PBMCs of patients with MG. Furthermore, OIP5-AS1 negatively modulated the expression of miR-181c-5p in Jurkat cells. Additionally, the results of the luciferase reporter assay confirmed that miR-181c-5p was a direct target of OIP5-AS1. Collectively, OIP5-AS1 participated in MG progression by sponging miR-181c-5p.

IL-7 is a pivotal cytokine that plays a key role in the development of Tfh cells and the germinal centers of B cells (Corfe & Paige, 2012; Rivière et al., 2021; Seo et al., 2014). In addition, a previous study has demonstrated that IL-7 is a target of miR-181c-5p (Zhang et al., 2016). In this study, real-time PCR results showed an increase in IL-7 expression in MG patients. Furthermore, our findings revealed a negative correlation between IL-7 expression and miR-181c-5p and a positive correlation with OIP5-AS1 in PBMCs of MG patients. Therefore, we hypothesized that OIP5-AS1 acts as a ceRNA sponging miR-181c-5p to regulate IL-7 expression. Moreover, we found that the knockdown of OIP5-AS1 suppressed IL-7 mRNA and protein expression levels in Jurkat cells, while these changes were nullified by the miR-181c-5p inhibitor. These results indicate that OIP5-AS1 modulates the expression of IL-7 by sponging miR-181c-5p. Functional experiments suggested that OIP5-AS1 silencing suppressed proliferation and promoted apoptosis in Jurkat cells, whereas inhibition of miR-181c-5p reversed these effects.

However, this study contains some limitations, such as the small sample size used for OIP5-AS1 expression analysis of patients with MG. As such, this necessitates further studies with larger cohorts of patients with MG. Previous studies have found that miR-181c is downregulated in activated human peripheral blood CD4^+^T cells (Xue et al., 2011). However, in our study, specific cell types expressing OIP5-AS1 and miR-181c-5p were not investigated. In addition, the possible OIP5-AS1 up-regulation in T cells require further study. Likewise, the effects of OIP5-AS1 have not been verified *in vivo*, and animal experiments validating the effects of OIP5-AS1 should be conducted in future investigations. Moreover, our experimental design was based on the ceRNA theory, which excluded some miRNAs that were also regulated by OIP5-AS1.

## Conclusions

In summary, we demonstrated that the lncRNA OIP5-AS1 is up-regulated in patients with MG. LncRNA OIP5-AS1 acts as a ceRNA and increases the expression of IL-7 by sponging miR-181c-5p. Knockdown of OIP5-AS1 inhibits proliferation and promotes apoptosis of Jurkat cells by sponging miR-181c-5p. Altogether, our findings provide novel insights into MG treatment.

## Supplemental Information

10.7717/peerj.13454/supp-1 Supplemental Information 1 Full-length uncropped blots (Fig. 4).

10.7717/peerj.13454/supp-2 Supplemental Information 2 LncRNA OIP5-AS1 is up-regulated, and miR-181c-5p is downregulated in MG patients. (A) OIP5-AS1 expression was examined in MG patients and control subjects by real-time PCR. (B) miR-181c-5p expression was examined in MG patients and control subjects by real-time PCR. (C) Pearson’s correlation was performed to analyze the correlation between OIP5-AS1 and miR-181c-5p expression in PBMCs of MG patients. The experiment was repeated at least three times, and the data are presented as the mean ± SD. *p < 0.05, **p < 0.01, ***p < 0.001.

10.7717/peerj.13454/supp-3 Supplemental Information 3 OIP5-AS1 is a direct target of miR-181c-5p. (A) The transfection efficiency of miR-181c-5p mimics and miR-181c-5p inhibitor were measured by real-time PCR. (B) The relative expression level of OIP5-AS1 in Jurkat cells transfected with miR-181c-5p mimics or miR-181c-5p inhibitor was measured using real-time PCR. (C) The expression of OIP5-AS1 in Jurkat cells transfected with shRNA against OIP5-AS1 (shRNA-1, shRNA-2, and shRNA-3), or negative control (shRNA NC) was measured using real-time PCR. The shRNA-1 with better knockdown efficiency was used for subsequent experiment, named shOIP5-AS1. (D) The relative expression level of miR-181c-5p in Jurkat cells transfected with shRNA NC or shOIP5-AS1 was measured using real-time PCR. (E) The putative miR-181c-5p binding sequence of the wide-type and mutation sequence of OIP5-AS1. (F) The luciferase reporter plasmid containing OIP5-AS1-WT or OIP5-AS1-MUT was co-transfected with miR-181c-5p mimics or miRNA NC into HEK293T cells. The experiment was repeated at least three times, and the data are presented as the mean ± SD.*p < 0.05, **p < 0.01, ***p < 0.001. ns, no significant.

10.7717/peerj.13454/supp-4 Supplemental Information 4 OIP5-AS1 regulates IL-7 expression by sponging miR-181c-5p in a ceRNA manner. (A) IL-7 expression was examined in MG patients and control subjects by real-time PCR. (B) Pearson’s correlation was performed to analyze the correlation between IL-7 and miR-181c-5p expression in PBMCs of MG patients. (C) Pearson’s correlation was performed to analyze the correlation between IL-7 and OIP5-AS1 expression in PBMCs of MG patients. (D) Relative expression levels of IL-7 mRNA were measured by real-time PCR after transfection with miR-181c-5p mimic or miRNA NC in Jurkat cells. (E) Relative expression levels of IL-7 protein were measured by western blotting after transfection with miR-181c-5p mimic or miRNA NC in Jurkat cells. (F) Relative expression levels of IL-7 mRNA were measured by real-time PCR after transfection with shRNA NC + miR-181c-5p inhibitor NC, shOIP5-AS1 + miR-181c-5p inhibitor NC, and shOIP5-AS1 + miR-181c-5p inhibitor in Jurkat cells. (G) Relative expression levels of IL-7 protein were measured by western blotting after transfection with shRNA NC + miR-181c-5p inhibitor NC, shOIP5-AS1 + miR-181c-5p inhibitor NC, and shOIP5-AS1 + miR-181c-5p inhibitor in Jurkat cells. The experiment was repeated at least three times, and data are presented as the mean ± SD. *p < 0.05, **p < 0.01, ***p < 0.001. ns, no significant.

10.7717/peerj.13454/supp-5 Supplemental Information 5 OIP5-AS1 inhibits apoptosis and promotes cell proliferation by sponging miR-181c-5p. (A) Flow cytometry was performed to determine cell apoptosis after transfection with shRNA NC + miR-181c-5p inhibitor NC, shOIP5-AS1 + miR-181c-5p inhibitor NC, and shOIP5-AS1 + miR-181c-5p inhibitor for 48 h into Jurkat cells. (B) The apoptosis rate of Jurkat cells after transfection with shRNA NC + miR-181c-5p inhibitor NC, shOIP5-AS1 + miR-181c-5p inhibitor NC, and shOIP5-AS1 + miR-181c-5p inhibitor. (C) CCK-8 assay was performed to determine cell proliferation after transfection with shRNA NC + miR-181c-5p inhibitor NC, shOIP5-AS1 + miR-181c-5p inhibitor NC, and shOIP5-AS1 + miR-181c-5p inhibitor into Jurkat cells. The experiment was repeated at least three times, and data are presented as the mean ± SD. *p < 0.05, **p < 0.01, ***p < 0.001. ns, no significant.

## Additional Information and Declarations

### Data Availability

The following information was supplied regarding data availability: The raw data are available in the Supplemental Files.

## References

[ref-1] Batista PJ, Chang HY (2013). Long noncoding RNAs: cellular address codes in development and disease. Cell.

[ref-2] Bi X, Guo XH, Mo BY, Wang ML, Luo XQ, Chen YX, Liu F, Olsen N, Pan YF, Zheng SG (2019). LncRNA PICSAR promotes cell proliferation, migration and invasion of fibroblast-like synoviocytes by sponging miRNA-4701-5p in rheumatoid arthritis. EBioMedicine.

[ref-3] Çebi M, Durmus H, Aysal F, Özkan B, Gül GE, Çakar A, Hocaoglu M, Mercan M, Yentür SP, Tütüncü M, Yayla V, Akan O, Dogan Ö, Parman Y, Saruhan-Direskeneli G (2020). CD4(+) T cells of myasthenia gravis patients are characterized by increased IL-21, IL-4, and IL-17A productions and higher presence of PD-1 and ICOS. Frontiers in Immunology.

[ref-4] Chen YG, Satpathy AT, Chang HY (2017). Gene regulation in the immune system by long noncoding RNAs. Nature Immunology.

[ref-5] Cheng Z, Qiu S, Jiang L, Zhang A, Bao W, Liu P, Liu J (2013). MiR-320a is downregulated in patients with myasthenia gravis and modulates inflammatory cytokines production by targeting mitogen-activated protein kinase 1. Journal of Clinical Immunology.

[ref-6] Corfe SA, Paige CJ (2012). The many roles of IL-7 in B cell development; mediator of survival, proliferation and differentiation. Seminars in Immunology.

[ref-7] Cortés-Vicente E, Gallardo E, Martínez M, Díaz-Manera J, Querol L, Rojas-García R, Illa I (2016). Clinical characteristics of patients with double-seronegative myasthenia gravis and antibodies to cortactin. JAMA Neurology.

[ref-8] Dai J, Xu L, Hu X, Han G, Jiang H, Sun H, Zhu G, Tang X (2018). Long noncoding RNA OIP5-AS1 accelerates CDK14 expression to promote osteosarcoma tumorigenesis via targeting miR-223. Biomedicine and Pharmacotherapy.

[ref-9] Fatica A, Bozzoni I (2014). Long non-coding RNAs: new players in cell differentiation and development. Nature Reviews Genetics.

[ref-10] Gilhus NE (2016). Myasthenia gravis. New England Journal of Medicine.

[ref-11] Gilhus NE, Tzartos S, Evoli A, Palace J, Burns TM, Verschuuren J (2019). Myasthenia gravis. Nature Reviews Disease Primers.

[ref-12] Liang M, Wang H, Liu C, Lei T, Min J (2021). OIP5-AS1 contributes to the development in endometrial carcinoma cells by targeting miR-152-3p to up-regulate SLC7A5. Cancer Cell International.

[ref-13] Liu R, Li Y, Zhou H, Wang H, Liu D, Wang H, Wang Z (2021). OIP5-AS1 facilitates Th17 differentiation and EAE severity by targeting miR-140-5p to regulate RhoA/ROCK2 signaling pathway. Life Sciences.

[ref-14] Liu XH, Sun M, Nie FQ, Ge YB, Zhang EB, Yin DD, Kong R, Xia R, Lu KH, Li JH, De W, Wang KM, Wang ZX (2014). Lnc RNA HOTAIR functions as a competing endogenous RNA to regulate HER2 expression by sponging miR-331-3p in gastric cancer. Molecular Cancer.

[ref-15] Long H, Wang X, Chen Y, Wang L, Zhao M, Lu Q (2018). Dysregulation of microRNAs in autoimmune diseases: pathogenesis, biomarkers and potential therapeutic targets. Cancer Letters.

[ref-16] Luo M, Liu X, Meng H, Xu L, Li Y, Li Z, Liu C, Luo YB, Hu B, Xue Y, Liu Y, Luo Z, Yang H (2017). IFNA-AS1 regulates CD4(+) T cell activation in myasthenia gravis though HLA-DRB1. Clinical Immunology.

[ref-17] Mantegazza R, Bernasconi P, Cavalcante P (2018). Myasthenia gravis: from autoantibodies to therapy. Current Opinion in Neurology.

[ref-18] Niu L, Jiang J, Yin Y, Hu B (2020). LncRNA XLOC_003810 modulates thymic Th17/Treg balance in myasthenia gravis with thymoma. Clinical and Experimental Pharmacology and Physiology.

[ref-19] Rivière E, Pascaud J, Virone A, Dupré A, Ly B, Paoletti A, Seror R, Tchitchek N, Mingueneau M, Smith N, Duffy D, Cassard L, Chaput N, Pengam S, Gauttier V, Poirier N, Mariette X, Nocturne G (2021). Interleukin-7/Interferon axis drives T cell and salivary gland epithelial cell interactions in Sjögren’s syndrome. Arthritis & Rheumatology.

[ref-20] Romi F, Gilhus NE, Aarli JA (2005). Myasthenia gravis: clinical, immunological, and therapeutic advances. Acta Neurologica Scandinavica.

[ref-21] Seo YB, Im SJ, Namkoong H, Kim SW, Choi YW, Kang MC, Lim HS, Jin HT, Yang SH, Cho ML, Kim YM, Lee SW, Choi YK, Surh CD, Sung YC (2014). Crucial roles of interleukin-7 in the development of T follicular helper cells and in the induction of humoral immunity. Journal of Virology.

[ref-22] Shi C, Yang Q, Pan S, Lin X, Xu G, Luo Y, Zheng B, Xie X, Yu M (2020). LncRNA OIP5-AS1 promotes cell proliferation and migration and induces angiogenesis via regulating miR-3163/VEGFA in hepatocellular carcinoma. Cancer Biology & Therapy.

[ref-23] Statello L, Guo CJ, Chen LL, Huarte M (2021). Gene regulation by long non-coding RNAs and its biological functions. Nature Reviews: Molecular Cell Biology.

[ref-24] Tay Y, Rinn J, Pandolfi PP (2014). The multilayered complexity of ceRNA crosstalk and competition. Nature.

[ref-25] Vincent A, Palace J, Hilton-Jones D (2001). Myasthenia gravis. Lancet.

[ref-26] Wang J, Cao Y, Lu X, Wang X, Kong X, Bo C, Li S, Bai M, Jiao Y, Gao H, Yao X, Ning S, Wang L, Zhang H (2020a). Identification of the regulatory role of lncRNA SNHG16 in Myasthenia gravis by constructing a competing endogenous RNA network. Molecular Therapy - Nucleic Acids.

[ref-27] Wang J, Tang Q, Lu L, Luo Z, Li W, Lu Y, Pu J (2019). LncRNA OIP5-AS1 interacts with miR-363-3p to contribute to hepatocellular carcinoma progression through up-regulation of SOX4. Gene Therapy.

[ref-28] Wang W, Hu W, Wang Y, An Y, Song L, Shang P, Yue Z (2020b). Long non-coding RNA UCA1 promotes malignant phenotypes of renal cancer cells by modulating the miR-182-5p/DLL4 axis as a ceRNA. Molecular Cancer.

[ref-29] Wang Y, Lin C, Liu Y (2021). Molecular mechanism of miR-34b-5p and RNA binding protein HuR binding to lncRNA OIP5-AS1 in colon cancer cells. Cancer Gene Therapy.

[ref-30] Xue Q, Guo ZY, Li W, Wen WH, Meng YL, Jia LT, Wang J, Yao LB, Jin BQ, Wang T, Yang AG (2011). Human activated CD4(+) T lymphocytes increase IL-2 expression by downregulating microRNA-181c. Molecular Immunology.

[ref-31] Zhang Y, Guo M, Xin N, Shao Z, Zhang X, Zhang Y, Chen J, Zheng S, Fu L, Wang Y, Zhou D, Chen H, Huang Y, Dong R, Xiao C, Liu Y, Geng D (2016). Decreased microRNA miR-181c expression in peripheral blood mononuclear cells correlates with elevated serum levels of IL-7 and IL-17 in patients with myasthenia gravis. Clinical and Experimental Medicine.

