# Supplementary figures and images for: LncRNA OIP5-AS1 modulates the proliferation and apoptosis of Jurkat cells by sponging miR-181c-5p to regulate IL-7 expression in myasthenia gravis
Source: PeerJ. 2022 May 17;10:e13454. doi: 10.7717/peerj.13454 (PMC9121865; doi:10.7717/peerj.13454)

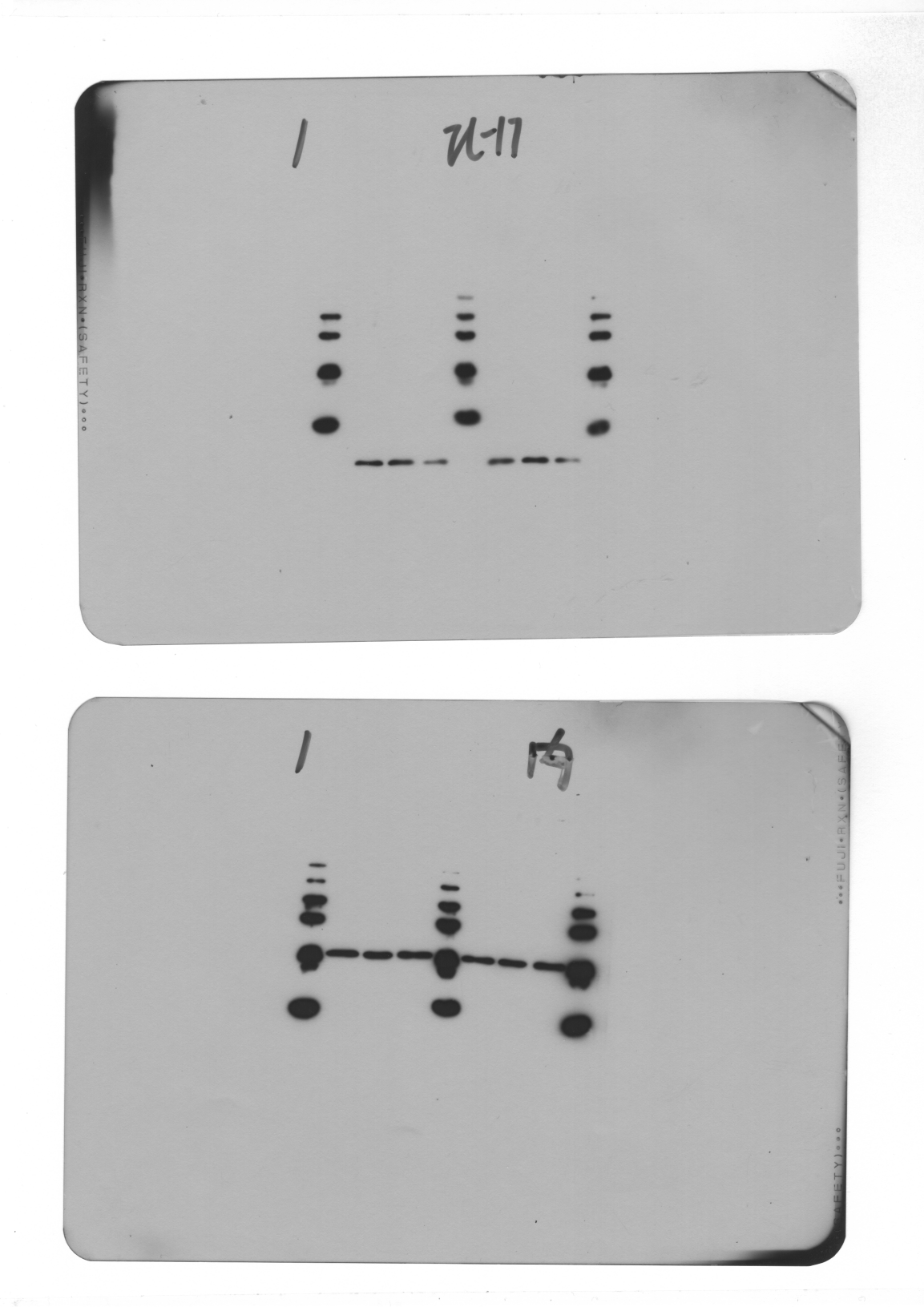


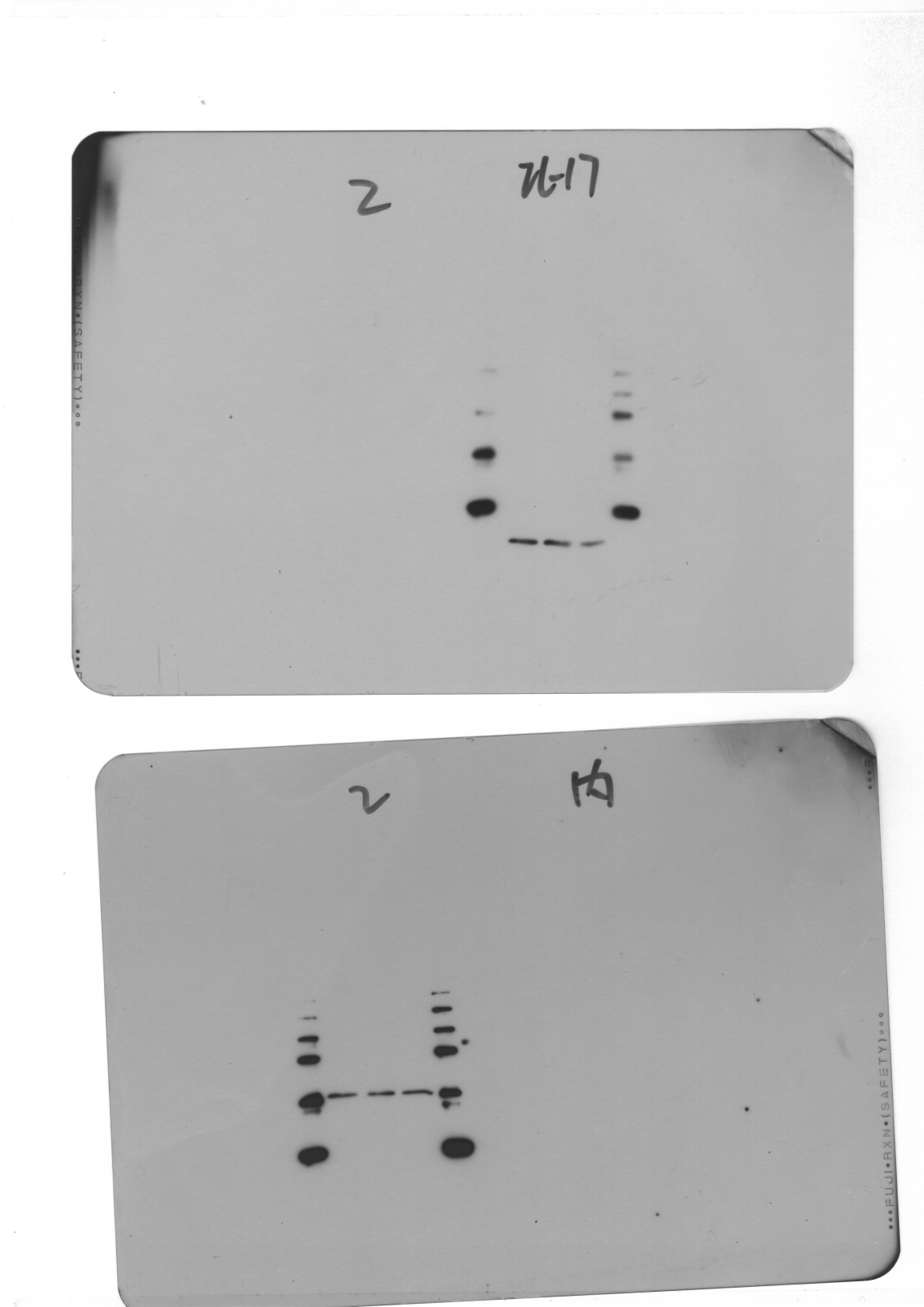


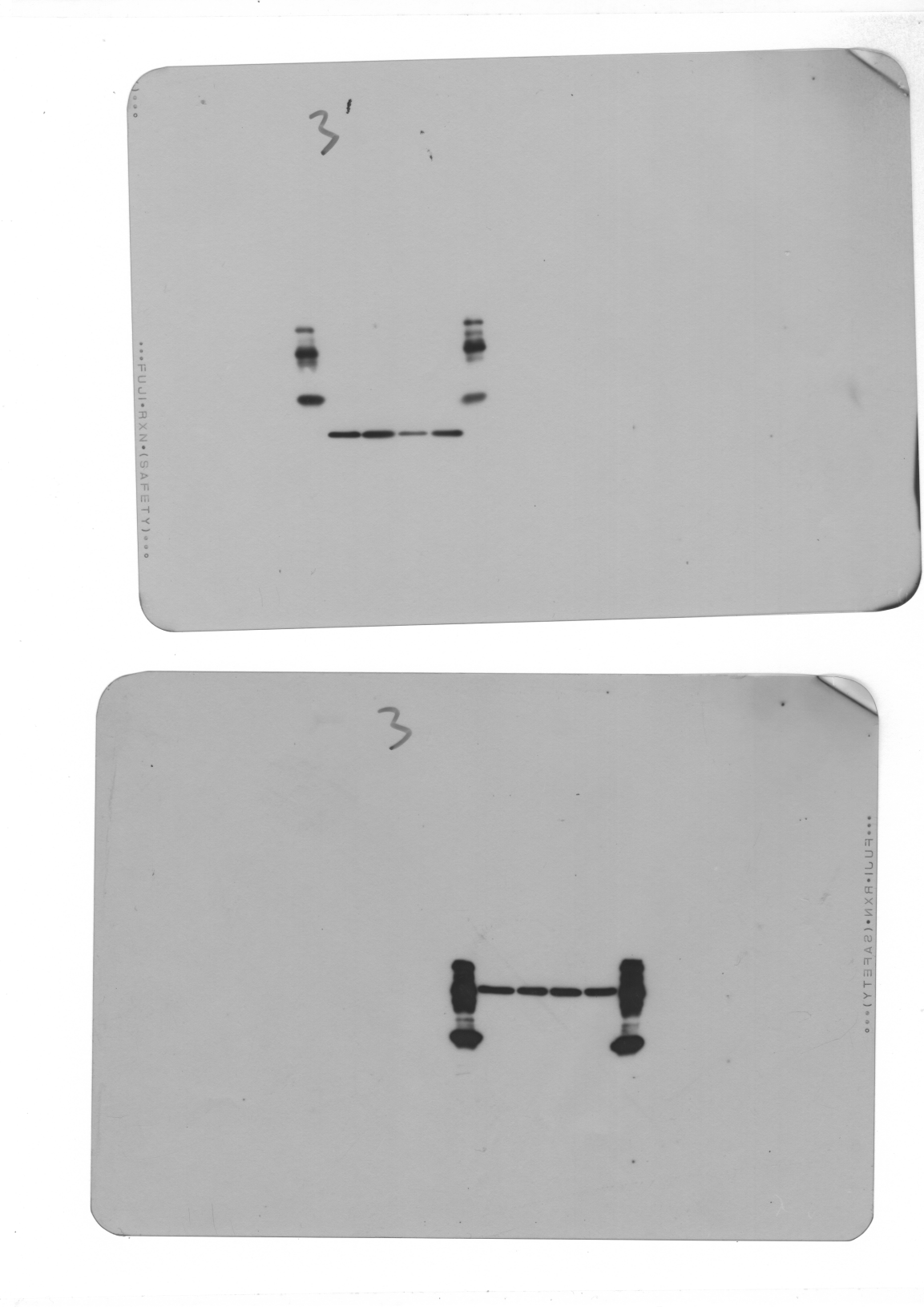


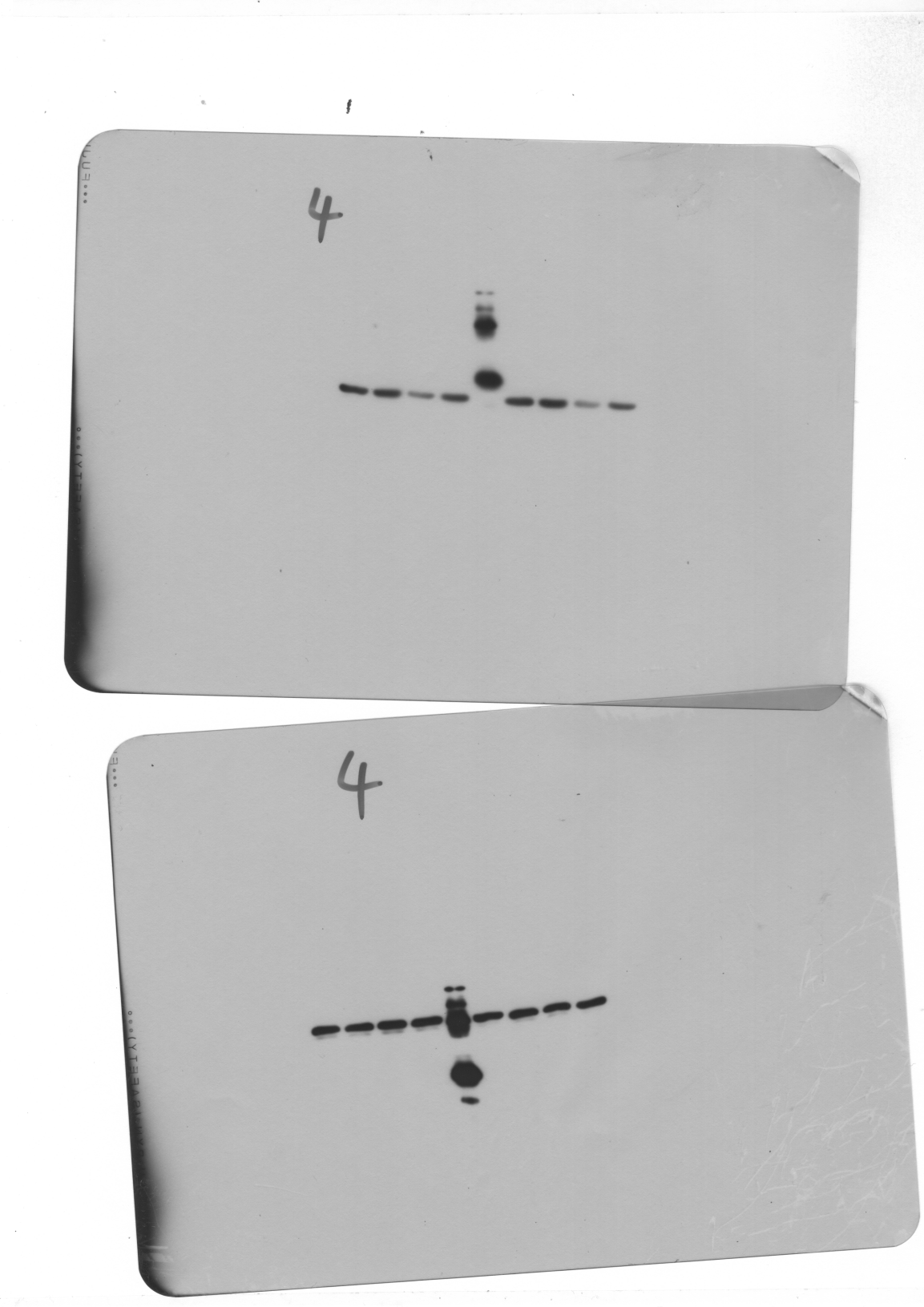

Supplement: Supplemental Information 1 [file peerj-10-13454-s001.docx]
